# Exact, time-independent estimation of clone size distributions in normal and mutated cells

**DOI:** 10.1098/rsif.2014.0654

**Published:** 2014-10-06

**Authors:** A. Roshan, P. H. Jones, C. D. Greenman

**Affiliations:** 1 MRC Cancer Cell Unit, Hutchison-MRC Research Centre, Cambridge CB2 2XZ, UK; 2 School of Computing Sciences, University of East Anglia, Norwich NR4 7TJ, UK; 3 The Genome Analysis Centre, Norwich Research Park, Norwich NR4 7UH, UK

**Keywords:** clone size distribution, Dyck paths, Motzkin triangle, Luria–Delbrück, mathematical modelling

## Abstract

Biological tools such as genetic lineage tracing, three-dimensional confocal microscopy and next-generation DNA sequencing are providing new ways to quantify the distribution of clones of normal and mutated cells. Understanding population-wide clone size distributions *in vivo* is complicated by multiple cell types within observed tissues, and overlapping birth and death processes. This has led to the increased need for mathematically informed models to understand their biological significance. Standard approaches usually require knowledge of clonal age. We show that modelling on clone size independent of time is an alternative method that offers certain analytical advantages; it can help parametrize these models, and obtain distributions for counts of mutated or proliferating cells, for example. When applied to a general birth–death process common in epithelial progenitors, this takes the form of a gambler's ruin problem, the solution of which relates to counting Motzkin lattice paths. Applying this approach to mutational processes, alternative, exact, formulations of classic Luria–Delbrück-type problems emerge. This approach can be extended beyond neutral models of mutant clonal evolution. Applications of these approaches are twofold. First, we resolve the probability of progenitor cells generating proliferating or differentiating progeny in clonal lineage tracing experiments *in vivo* or cell culture assays where clone age is not known. Second, we model mutation frequency distributions that deep sequencing of subclonal samples produce.

## 1. Introduction

One approach to understanding the cellular hierarchy in multicellular organized tissue has been tracking the fate of individual cells either labelled *in vivo* or isolated *ex vivo* [1–6]. Improved techniques, including genetic lineage tracing and three-dimensional imaging by confocal microscopy, have helped us further investigate this basic area of research and have rapidly become the gold standard approach [7–9]. Typically, a cell type of interest is labelled with an identifier, and the distribution of its progeny at later time points is observed. Clone distribution data can then be used to decipher division dynamics across the population of cells with great resolution. However, the current methods use population averaging, and are time-dependent posing analytical and technical challenges. There is thus a need for alternative statistical approaches that may be complementary.

**Figure 1.**
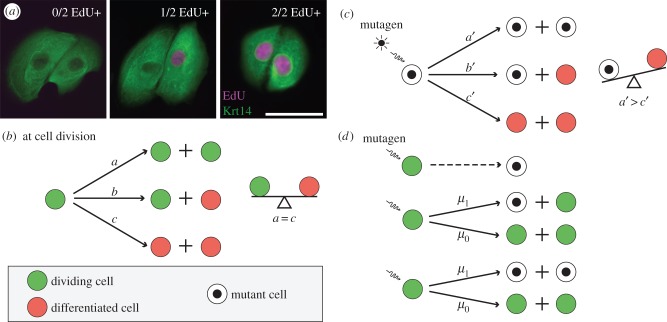
Colony formation in normal and mutated cells. (*a*) Immunofluorescence images of two-cell clones of cultured primary human keratinocytes stained with the keratinocyte marker keratin14, and the proliferation marker EdU, showing three possible outcomes of division: two non-proliferating daughters (0/2 EdU + ), a non-proliferating and a proliferating daughter (1/2 EdU+), or two proliferating daughters (2/2 EdU+). Scale bar, 50 µm. (*b*) Cell division is a birth–death process with three possible outcomes based on the proliferative ability of its daughters. As above, a dividing cell (P) may divide into two dividing daughters (PP), a dividing and differentiated daughter (PD), or two differentiated daughters (DD) in proportions *a*, *b* and *c,* respectively. In homeostatic tissues, the number of new dividing cells is equal to the number of non-dividing cells (*a* = *c*). (*c*) In the presence of mutagens such as UV radiation, this process is imbalanced in p53 mutant clones in favour of proliferation (*a’* > *c’*). This gives a survival advantage to mutant clones. (*d*) Mutant cell formation itself is a birth process that can follow one of three possibilities. The first is cell division independent and can occur with background exposure. The second and third possibilities occur following cell division, producing one or two mutant cells out of two daughter cells with probability *μ*_1_ = 1 − *μ*_0_.

Adult mammalian epithelium has a high rate of cell division during steady state. Despite this rapid rate of proliferation, the tissue remains in homeostasis as new cells are being generated at the same rate as loss of differentiated cells in a birth–death process (*a* = *c* in figure 1*b*). A simple illustration of this is in the interfollicular epidermis, where cell division occurs in the basal layer of a multi-layered epithelium. Cell division here can produce proliferating daughters, that remain in the basal layer, or non-dividing daughters, which are shed to the suprabasal layers, and eventually lost in a process of differentiation. When these keratinocytes are grown in culture, a typical cell division can result in two dividing daughters, one dividing daughter or no dividing daughter out of two total daughters as seen through the uptake of the proliferation marker 5-ethynyl-2′-deoxyuridine (EdU; figure 1*a*). Genetic lineage tracing in basal keratinocytes has allowed conditional expression of fluorescent proteins, with all subsequent daughter cells retaining the label, and thus being highlighted as a clone. The distribution of clone sizes will depend upon the relative rates of different outcomes of division (*a*, *b* and *c* in figure 1*b*) [1]. Reserve stem cells provide significant contribution during wound healing [3,10]. This balance is also disturbed in chronic UV irradiation, where p53 mutant keratinocyte clones gain a survival advantage over non-mutant clones mediated through increased proportions of proliferative daughters [11] (*a* > *c* in figure 1*c*). The recent technical advance of live imaging in epithelia may provide us additional information to these models, such as the distribution of cell cycle times [12].

One of the main problems for such systems is the estimation of the rates *a*, *b* and *c*. There are two current approaches. First, we can use direct microscopic observation. This involves the observation of many cells over several cell divisions. With a sufficient number of cell divisions, one can then examine the proportions of distinct classes of cell divisions to estimate these parameters. There are several factors that make this approach difficult. First, tracking cells over long periods of time is a complex and resource intensive task and more efficient methods are desirable. Second, different classes of cell (such as P and D) can be visually indistinguishable, and the only discerning characteristic is whether subsequent division occurs (implying a P cell). This makes identification of the three types of cell division associated with *a*, *b* and *c* difficult.

The second estimation approach is to relate the probabilities *a* and *c* to the subsequent clone size distribution of tagged cells. This approach requires sufficient time for the development of substantive clones, which will contain a mixture of differentiated and proliferating cells. This was implemented in [2] for example, where estimates of *a* = *c* = 0.1 ± 0.01 and *b* = 0.80 ± 0.02 were obtained. However, this approach involves months of clonal development and is sensitive to the loss of shedding differentiated cells from the suprabasal layer, which is difficult to quantify.

Both techniques highlight a desire for a method that can both circumvent some of these technical challenges and is relatively quick to implement. Now, a single labelled proliferating P cell left to divide *in vivo* will result in a *fully differentiated* clone of size *n* with some probability *p_n_*(*a*,*b*,*c*) that depends upon parameters *a*, *b* and *c*. In longer-term *in vivo* experiments, these clones will have entered the suprabasal layer and sloughed out of the system. We estimate these parameters from the observed distribution of fully differentiated clones. These clones are generally small and rapidly form, meaning the method is relatively quick. Because we are only using counts of clone sizes, it also circumvents the need to observe all cell divisions, resulting in a less intensive microscopy technique.

There is also an increasing body of work investigating the growth dynamics of pre-neoplastic and neoplastic tissue [13–17]. A growing colony of cells can be modelled as a branching process. Luria & Delbrück [18] were the first to produce an analytical examination of the distribution of the number of mutant cells in growing bacterial colonies. They used this to show that mutations arise randomly rather than in response to the environment. Their argument was partly deterministic, and Lea & Coulson [19] and Bartlett [20,21] derived approaches with greater stochastic rigour. These methods generally consider the problem of how many mutants are present after a fixed amount of time. An unpublished combinatorial method by Haldane also exists [22] where all cells divide simultaneously.

These distributions generally assign genes the binary status of mutated or non-mutated. They do not consider the number of distinct mutations in a gene, or the number of different combinations of mutations a subclone of cells may contain. Modern sequencing techniques mean greater resolution of mutations is now possible, and there is increased interest in considering distributions associated with combinations of mutations [23].

As Kendall observed [24,25], there are broadly three models for mutation formulation (figure 1*d*). The first formulation would indicate a single cell converts to mutated status at any time independent of the cell division process. This may be the case for continuous exposure to mutagens, such as UV light [26]. The second formulation is the most common formulation where mutations occur in one of the two daughter cells during the cell division process. This is likely to be the case for many mutational processes, where nucleotide errors occur on one of the two DNA strands [27]. DNA repair machinery then erroneously corrects this during checkpoints in the cell cycle, resulting in one mutant daughter cell. The third formulation assumes that both daughter cells are mutant. This is also a valid model, and is likely to arise when double-stranded breaks occur. When double-stranded repair incorrectly repairs the damage, rearrangements result and both daughter cells will be mutant. Some processes such as breakage–fusion–bridge cycles will even result in two mutant daughter cells with distinct rearrangements [28,29]. For analytical purposes in this paper, we assume the most common second formulation. Additionally, we assume that a mutation does not increase the chance of cell loss through apoptosis.

In this work, we consider a different statistical approach to clonal distributions. A standard technique to analysing a branching process involving two classes of objects, such as mutant/non-mutant, or progenitor/differentiated, is to write down a Chapman–Kolmogorov equation for *P_m_*_,*n*_(*t*); the probability of having *m* and *n* cells of the two types, at time *t*, and obtain a solution [30]. Instead, we determine the distribution of the number of different types of cells that are present when a fixed number of cells have accumulated, rather than the time that has passed. With this approach, we see that treating cell differentiation or mutation as time-independent results in exact analytic forms for the distributions of interest. In §2, we obtain the distribution for the number of dividing cells in an epithelial population. We then obtain distributions for the number of mutant cells in a clone undergoing a pure birth process.

## 2. Distribution of colony sizes in homeostatic tissue

Tissue homeostasis is balanced by two types of cells: progenitor (dividing) cells (P) and differentiated (non-dividing) cells (D). As progenitor cells (P) divide, they produce two daughter cells which may be either a progenitor cell or a differentiated cell (D) resulting in the combinations (PP), (PD) or (DD). We assume the probabilities of these occurring are *a*, *b* and *c*, respectively, represented in figure 1*b*. Across a population, these probabilities are assumed to be constant, holding the same values for any cell division that takes place at steady state. There is the possibility that apoptosis may form an additional component of this process. While one could incorporate this as an additional branch in the process of figure 1*b*, it is assumed negligible in the following analysis.

For the sake of simplicity, we assume that we start with a single dividing cell. We also assume the number of descendant cells can be observed, but that (P) and (D) cells cannot be distinguished. There are two problems we would like to consider. First, if we trace the lineage of a single cell, then we wish to determine the distribution of the number of progenitor (P) cells present. Second, the physical similarity between (P) and (D) cells without any protein markers make the parameters *a*, *b* and *c* difficult to directly measure. Thus, we would like a method to estimate them.

**Figure 2.**
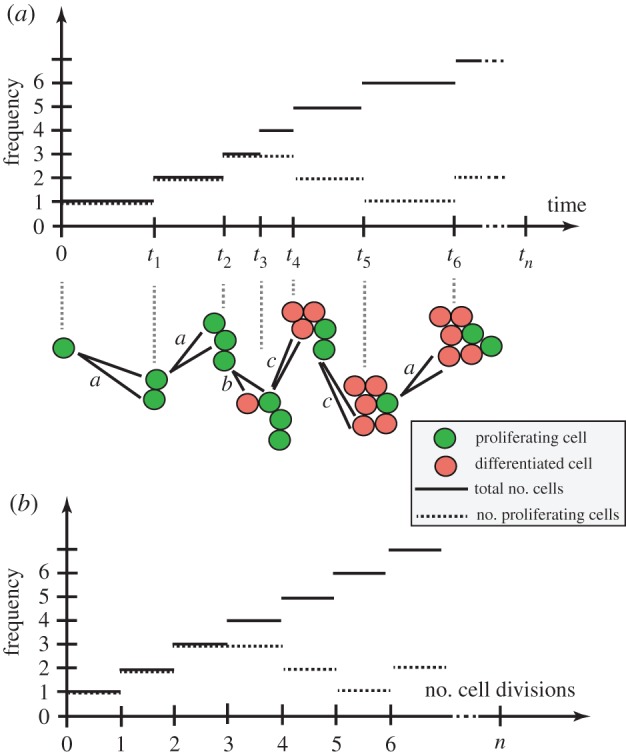
A branching process of differentiated and proliferating cells. A single dividing cell is followed in time with the height of the solid line indicating total number of cells, and the height of the dashed line indicating number of dividing cells. In (*a*), plotted against time, we see the rate of cell division is dependent upon the number of proliferating cells. In (*b*), plotted against number of cell divisions, we see the number of proliferating cells only depends upon the nature and number of cell divisions, not their timing.

Now, our approach is based on the size of the clone (rather than time passed). Now, with each cell division, irrespective of outcome, the colony size *n* increases by 1 forming a clone of *n* + 1 cells. If the cell division results in two progenitor daughters (PP), the number of dividing cells *k* increases to *k* + 1. If the cell division results in a progenitor cell and a differentiated cell (PD), the number of dividing cells *k* stays the same. The production of two differentiated daughters (DD) results in a loss of dividing cells to *k* − 1. We can thus model the number of P cells as a discrete random walk that can move up, remain flat or move down with probabilities *a*, *b* and *c*, where we have one forward step to take at every cell division as in figure 2*a,b*. Note that if the colony becomes fully differentiated, *k* = 0, we have no dividing cells and our process stops.

We note that the timing of these divisions does not relate to the count of proliferating cells. In figure 2*a*, we see the time-dependent process, with a division rate that will be proportional to the number of proliferating cells. In figure 2*b*, we see the same information indexed by the number of cell divisions; the timing is not important. By restricting the stochastic process to the precise moments when the stochastic variable changes value, we have identified the *embedded Markov chain*. This may also be referred to as the *jump chain*, and the times between the *holding process* [31]. This is an intuitive technique that can be applied to discrete processes continuous in time, and was first employed by Kendall [32] to analyse queues. However, it does not appear to have been extensively used in clonal dynamics.

Such a problem is closely related to counting Motzkin lattice paths [33]. Lattice paths are paths connecting positions with integer coordinates and can take a variety of forms [34,35]. In particular, Motzkin paths start from the origin (0, 0) on a two-dimensional integer lattice and allow movement with an *up* (1, 1) step, a *flat* (1, 0) step or a *down* (1, −1) step such that we never move below the horizontal axis. There are several path counting techniques for such conditions [33,36,37], which have also seen applications to paths similar to the ones we describe [38,39]. These have been studied for a range of combinatorial problems [40], including some problems with weighted edges [41].

These paths can be used to represent our problem. The position (*n*, *k*) corresponds to the total number of cells, *n*, and the number of dividing cells, *k*, respectively. The PP, PD or DD divisions correspond to the up, flat and down steps, respectively. There are three differences from Motzkin paths to note. First, we start with one (P) cell, represented by position (1, 1). Second, we stop if we touch the horizontal axis, because no dividing (P) cells remain (*k* = 0). Lastly, we have probabilities *a*, *b* and *c* associated with each step. Now, we would like to find the probability *P_n_*,*_k_* of finding *k* dividing cells in a clone of size *n*. This probability then corresponds to a weighted sum of Motzkin paths from (1, 1) to (*n*, *k*), where Motzkin paths in this context do not touch the horizontal axis.

### 2.1. Motzkin paths describe the entire distribution of colony sizes

We have the following distribution for the number of progenitor (P) cells in a colony.

**Theorem 2.1.**

*If we seed a single dividing cell, then the probability of having k*(*>1*) *dividing cells when the colony is of size n is given by*
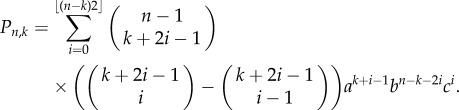

**Figure 3.**
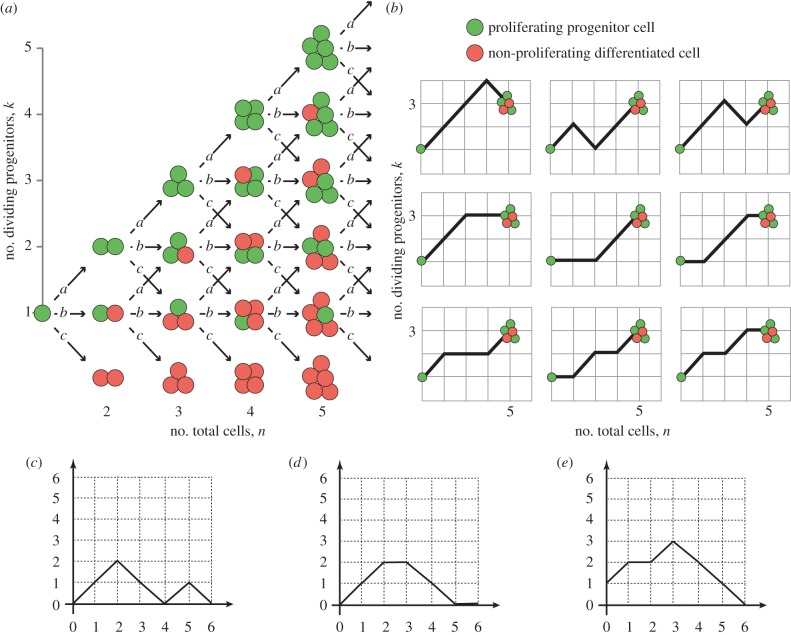
Cell proliferation as a combinatorial branching process with predictable paths. (*a*) Cell division in progenitor cells is a branching process with three possible outcomes (PP, PD or DD; figure 1). The expansion of a single cell to form a clone of cells is thus a combinatorial process, where any outcome of total clone size *n* and proliferating cells within it *k* occurs along fixed paths of a Motzkin-like triangle. Clones that reach the horizontal axis have only non-dividing cells, and therefore do not progress further. (*b*) Example showing the nine paths that a single proliferating cell can take to reach a clone of *n* = 5 and *k* = 3. The first three routes have three *a* divisions and one *c* division, whereas the remaining six routes involve two each of *a* and *b* divisions (cumulative probability = 3*a*^3^*c* + 6*a*^2^*b*^2^). (*c*) A Dyck path, which moves up and down and not below the horizontal axis. (*d*) A Motzkin path, which also includes horizontal moves. (*e*) A gambler's ruin problem, which starts from height 1 rather than from the origin, representing the formation of a fully differentiated clone from a single dividing cell.

*Proof*. We start with Dyck paths: paths from (0,0) to (0,2*n*) that do not go below the horizontal axis involving steps of type up, (1, 1), or down, (1, −1), such as portrayed in figure 3*a*. The number of such paths is known to be counted by the Catalan numbers 
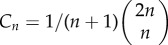
 [42]. A Dyck triangle is the collection of paths from (0,0) to (*n*, *k*) that do not go below the horizontal axis and involve up and down steps. Note that *n* and *k* must have the same parity. If *D_n_*_,*k*_ count these paths then conditioning over one step we find *D_n_*_,*k*_ = *D_n__−_*_1,*k*_*_−_*_1_ + *D_n__−_*_1,*k*__+__1_. It is straightforward to show by substitution that 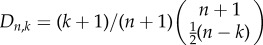 satisfies this recurrence, along with boundary condition *D*_2*n*,0_ = *C_n_*. This formula differs from other counts involving Dyck triangles, because this lattice formulation of the triangle is rotated through *π*/4 to the usual presentation [43].

We now turn to Motzkin paths, which are the same as Dyck paths except we now allow an additional horizontal step (1,0). Now, any Motzkin path from (0, 0) to (*n*, *k*) can be partitioned into a Dyck path from (0,0) to (*k* + 2*i*, *k*) involving *k* + *i* up steps and *i* down steps, along with *n* − *k* − 2*i* horizontal steps, where 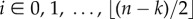. For any *i*, the probability of such a path arising is *a^k^*^+^ *^i^b^n^* *^−k^* *^−^*^2*i*^*c^i^*. Then, noting that we have 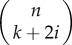 permutations of the horizontal steps with the Dyck path steps, we sum across the possibilities to get the following probability:
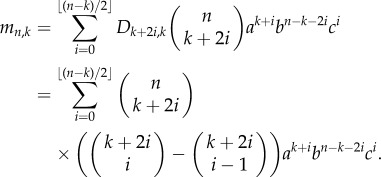

Finally, we note that we are going from position (1, 1) to (*n*, *k*) without touching the horizontal axis, so substituting *n* → *n* − 1 and *k* → *k* − 1 gives the required result: *P_n_*_,*k*_ = *m_n−_*_1,*k*_*_−_*_1_. ▪

This result allows us to look at the case where all *n* cells in the colony are fully differentiated (all are (D) cells), and there is not further potential for growth. In our Motzkin triangle analogy, this would be a Motzkin path (with an additional final down step) from (1, 1) to (*n*, 0), such as the path in figure 3*e*. All colonies that have a corresponding path touching the horizontal axis thus have no proliferating cells. We have an absorbing barrier, also known as the gambler's ruin problem.

**Corollary 2.1.**

*The probability P_n,0_ is given by weighted Motzkin numbers*
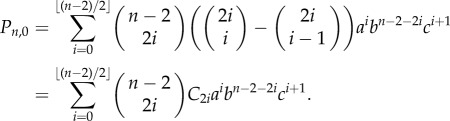

*Proof.* For the case where there are no dividing cells remaining in the colony, the colony must transit through a penultimate stage (*n* − 1, 1) with only one dividing cell remaining, and undergo an enforced final (DD) division. Multiplying the formula for *P_n_*_− 1,1_ by *c* gives the required result. ▪

Both these results have corresponding generating functions as described in the following result.

**Theorem 2.2.**

*The generating function* 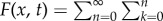 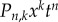 *is given by*
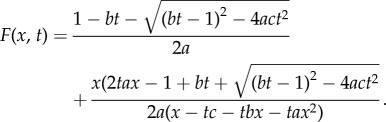

*Proof.* First, we construct a weighted generating function for paths in a standard Motzkin triangle, 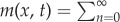 
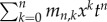
, where *m_n_*,*_k_* are the Motzkin numbers weighted by the elements *a*, *b* and *c* associated with each path from (0,0) to (*n*,*k*). Now, conditioning over a single step gives the following recurrence: *m_n_*_+1,k_ = *cm_n_*_,*k* +1_ + *bm_n_*_,*k*_ + *am_n_*_,*k* −1_. Then, substituting this into the generating function yields the following:
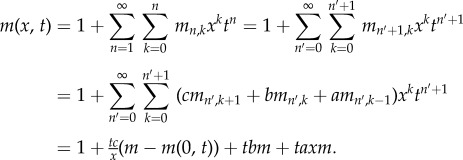

Rearranging this equation for *m*(*x*, *t*) results in the expression
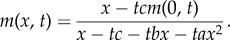

To find *m*(*0*, *t*), we note that a Motzkin path from (0, 0) to (*n* + 1, 0) involves one of two possible combinations. First, we can have an initial horizontal step (weight *b*) followed by a weighted Motzkin path of length *n*. Second, we can have an up step (weight *a*), a Motzkin path (length *k*), a down step (weight *c*) and a Motzkin path (length *n* − 1 − *k*). This is summarized in the following, where *m_n_* is the weighted sum of these paths:
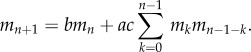

Now, substituting this recurrence into the generating function 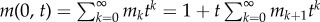 yields *m*(0, *t*) = 1 + *btm*(0, *t*) + *t*^2^*acm*(0, *t*)^2^. The solution satisfying *m*(0, 0) = 1 is then
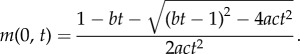

Substituting this into the equation for *m*(*x*, *t*) above then yields the general form
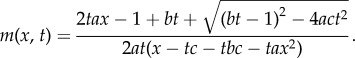

The result is obtained by noting that the generating function for *P_n_*_,*k*_ corresponds to paths from (1, 1) to (*n*, *k*). Furthermore, a path from (1, 1) to (*n*, 0) involves a weighted Motzkin path of length *n* − 2, followed by a down step, and we find that
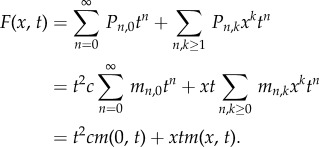

Substituting the weighted Motzkin generating functions results in the desired form. ▪

### 2.2. Gambler's ruin

We are now in a position to describe the probability of ruin, or equivalently the probability of a fully differentiated clone, where we have the following result.

**Corollary 2.2.**

*The generating function* 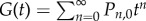 *is given by*
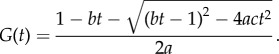

*This results in an alternative expression for the probability P_n_*_,0_ *that a clone of size n is fully differentiated:*
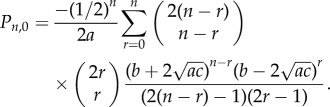

*Furthermore, we find that the probability P*_0_ *that a single proliferating cell will become fully differentiated is given by*
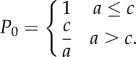

*Proof.* To obtain the generating function *G*(*t*), we simply substitute *x* = 0 into *F*(*x*, *t*) from theorem 2.2. To obtain the alternative expression for the probabilities *P_n_*_,0_ note that we can write *G*(*t*) as
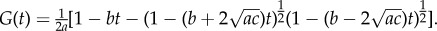

A double binomial expansion gives us
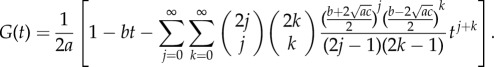

The constant and linear terms cancel, and a reordering of the summation to collect powers of *t* leaves us with the required expression.

Last, we note that 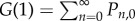 and so substituting *t* = 1 into the generating function gives us
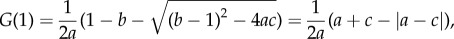
where we have used 1 − *b* = *a* + *c*. Separately considering the cases *a* > *c* and *a* ≤ *c* gives the required results.▪

### 2.3. Estimating differentiation probabilities

We are now in a position to estimate the probabilities *a*, *b* and *c* of getting the different daughter cell combinations of (PP), (PD) or (DD), even when (P) and (D) cells are visually indistinguishable. Clone size distributions in a range of homeostatic epithelia demonstrate that dividing progenitor cells have (PP) outcomes in similar proportions to (DD) outcomes (or *a* = *c*) [11]. Colonies arising from such populations will eventually become fully differentiated and stop growing, as represented in the bottom row of figure 3*a*. Therefore, at late time points of observation, all colonies of cells with few cell numbers will be formed exclusively of non-dividing cells, as any colonies with dividing cells will continue to expand in cell number. Thus, repeated measurements of small clone sizes, *n*_c_, of fully differentiated non-dividing colonies of size *n* can readily be counted. We can then compare these counts with the probabilities {*c*, *bc*, *c*(*b*^2^ + *ac*),…} = {*P_n_*_,0_}*_n_* of either corollary 2.2 or 2.1, and hence determine *a*, *b* and *c*.

We investigated this approach on triplicated sets of 7 day clonal cultures of human neonatal keratinocytes [44]. These cells divide faster than once per day, and at this time point, there is no shedding of differentiated cells, allowing us to apply our analysis. From a total population of 2086 keratinocyte clones, we observed 259, 72 and 53 colonies with two, three and four cells, respectively. Taking the ratios, we found that 72*/*259 = *bc/c* and 53*/*72 = *c*(*b*^2^ + *ac*)*/bc* which provided estimates *b* = 0.278 and *ac* = 0.127. The presence of additional proliferating clones was indicative of a skewed rate *a* > *c*. Noting that *b* = 1 − *a* − *c* finally produces estimates [*a*, *b*, *c*] = [0.415, 0.278, 0.307].

Small clone sizes form the bulk of clones seen in population distributions, and have therefore provided robust quantifiable results at early time points. It is also important to highlight that this analysis is not affected by the presence of additional cell populations which have a branching birth process alone (putative stem cell populations). Compared with the small, differentiated and non-expanding small clones, putative stem cell clones will be much larger, and continue to expand with time, thus being easily identified and excluded.

### 2.4. Stochastic processes approach

Finally, we remark that a lot of the derivations using Motzkin paths can also be replaced with approaches from stochastic processes. We highlight this with an alternative derivation of the gambler's ruin generating function of corollary 2.2 in appendix A.

## 3. Exact distributions of Luria–Delbrück type

We now investigate the mutation process of a growing clone of cells. Here, we assume no death process is involved, and initially that the mutation provides no additional survival advantage. In all that follows, *k* = *m* + *n* is the number of cells, where *m* and *n* count the number of mutants and non-mutants, respectively. Some aspects of this time-independent approach have been explored in [45], which we highlight when relevant.

### 3.1. The neutral model

**Figure 4.**
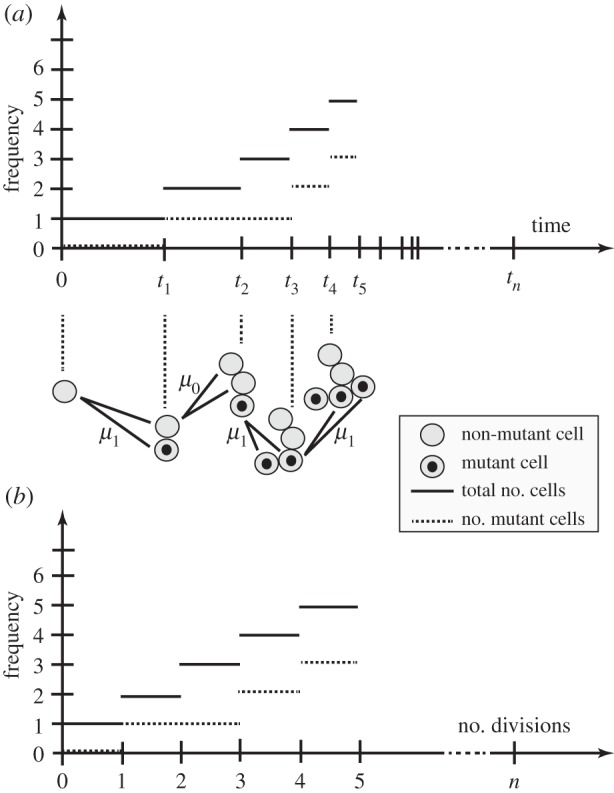
A branching process of non-mutated and mutated cells. A single dividing cell is followed in time with the height of the solid line indicating total number of cells, and the height of the dashed line indicating number of mutant cells. In (*a*), plotted against time, we see the rate of cell division is proportional to the total number of cells, resulting in exponential growth. In (*b*), plotted against the number of divisions, we see the number of mutant cells only depends upon the number of the mutant cell divisions not their timing.

Again, we start with a single dividing cell. An example of this can be seen in figure 4*a*. The cells are dividing randomly at a rate *β* according to the following Markovian branching (Yule–Furry) process. When any non-mutant cell divides we assume a mutant cell arises with probability *μ*_1_, such as the first division of figure 4*a* at time *t*_1_. Conversely, we may obtain two non-mutants with probability *μ*_0_ = 1 − *μ*_1_, such as in the second division portrayed at time *t*_2_. Finally, any dividing mutant produces two mutant daughters with probability 1, as displayed at times *t*_3_ and *t*_4_. We ignore any back mutation or loss of mutation.

As the colony grows, the rate of division, *βk*, increases in proportion to the number of cells present, *k*. If *t_k_* is the time of the *k*th division, then the mean time intervals *t_k_*_+ 1_ − *t_k_* correspondingly decrease as we get exponential growth. Note that at time *t_k_* the colony increases in size (by one cell) to *k* + 1 cells. It is this single dividing cell that has the opportunity to affect the number of mutations at this point; this is independent of either the time *t_k_* at which this takes place, or the time *t_k_*_+ 1_ − *t_k_* between divisions. We thus find we are interested in the embedded Markov (or jump) chain of the process, which proved so useful in the last section [31].

In figure 4*b*, we see the mutation process as a discrete process on the number of divisions that have taken place. We assume for the moment that mutant and non-mutant cells divide at the same rate in a Markovian manner. All cells are thus equally likely to divide at any point in time. If we have *n* non-mutant cells and *m* mutant cells, we then find that a mutant will divide with probability *m*/(*m* + *n*) resulting in *m* + 1 mutants and *m* + *n* + 1 cells. Conversely, a non-mutant divides with probability *n*/(*m* + *n*) resulting in *m* + *n* + 1 cells. This non-mutant will mutate with probability *μ*_1_ resulting in *m* + 1 mutants; otherwise, we will still have *m* mutants, with probability *μ*_0_. Then, conditioning over a single cell division leads to the following correspondence.

**Theorem 3.1 (Angerer [45]).**

*If* 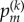 *denotes the probability of having m mutant cells present when the population is of size k, then we have the following recurrence, which is initialized with 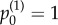:*
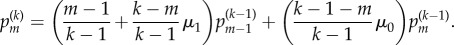

Note that we have reduced the mutation process to a discrete heterogeneous Markovian random walk starting from (0, 1) where we have either a horizontal step (1, 0) with probability ((*k* − *m*)/*k*)*μ*_0_, or the step (1, 1) with probability *m/k* + ((*k* − *m*)/*k*)*μ*_1_. In the next result, we describe the following general form for the *k* division distribution of mutants and non-mutants. These probabilities are simpler to express in terms of the non-mutants; 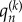. We also provide a corresponding generating function. This generalizes [45] slightly and provides a constructive proof of the formula for 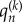, which is just validated by induction in [45], giving little insight into its derivation.

**Theorem 3.2.**

*The probability* 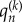 *of n non-mutant cells among k cells, starting from a single non-mutant cell is*
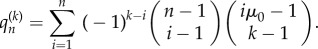

*These probabilities have the following generating function:*
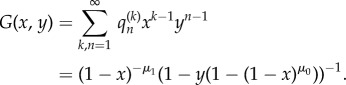

*Proof.* We rearrange the recurrence of theorem 3.1 in terms of non-mutants to give
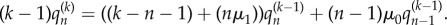

Multiplying by *x*^*k* −1^*y^n −^*^1^ and summing results in the following partial differential equation:
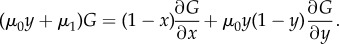

Note that conserved total probability is equivalent to boundary condition *G*(0, *y*) = 1. We then solve this with the method of characteristics to give the form stated for *G*. Three binomial expansions results in a power series in *x*,*y* with coefficients equal to the expression given for 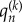. ▪

**Figure 5.**
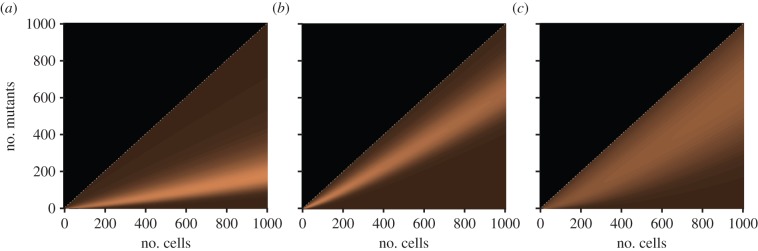
The distributions for the number of mutants for a range of colony sizes up to 1000 cells. (*a*) For *μ*_1_ = 0.05, *ρ* = 1, (*b*) For *μ*_1_ = 0.2, *ρ* = 1, (*c*) For *μ*_1_ = 0.05, *ρ* = 2, where *μ* is the mutation rate and *ρ* is the relative mutant fitness (*μ*_1/_*μ*_0_). (Online version in colour.)

An example of the resulting distributions can be seen in figure 5*a*,*b*.

We note that we have the zero mutant probability 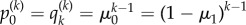, reflecting the requirement that all *k* − 1 divisions are mutant free. We can compare this with the classic result of Luria–Delbrück, which states that *p*_0_ = *e^−m^*, where *m* is the mean number of mutations. Now, this is simply the per cell division rate, *μ*_1_, multiplied by the number of divisions, *k* − 1, and we obtain *p*_0_ = *e*^−*μ*_1_^^(*k*^*^−^*^1)^ = (*e*^−*μ*_1_^)*^k−^*^1^. Now, *e*^−*μ*_1_^≈1 − *μ*_1_ and the two forms agree up to 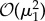.

We have the following result concerning the moments.

**Theorem 3.3.**

*If* 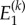 *and* 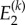 *represent the first two moments of the distribution of the number of non-mutants, conditional upon k cells being present, then we have the following results:*
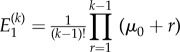
and
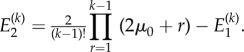

*Proof.* Differentiating the definition and functional form of *G* in theorem 3.2 gives us
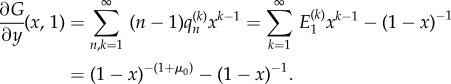

A series expansion then provides the form for the first moment.

The second moment is obtained similarly from 
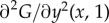
. ▪

### 3.2. Incorporating selection

For certain mutations, there may be a subsequent growth advantage. This has been observed with p53 mutations in epidermal tissue, for example [11]. Our assumption that all cells are equally likely to divide is no longer valid, with mutants dividing at a different rate from non-mutants. However, we find that the mutation process is only dependent upon the ratio of these rates, and we can condition on the number of cells and apply a similar technique to the previous section to obtain the following.

**Theorem 3.4.**

*Let the division rate for non-mutants and mutants be *β*_n_ and *β*_m_, respectively, with ratio *ρ* = *β*_m_/*β*_n_. If* 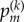 *represents the probability of having m mutant cells when there are k = m + n cells present, then we have the following recurrence, initialized with 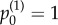:*
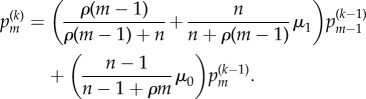

*Proof.* We suppose that the mutant cells are dividing at a rate *β_m_* and the non-mutant cells are dividing at a rate *β_n_*. We further suppose we have *m* and *n* of these cells, respectively. Then, if *T_m_* is the time until the next mutant cell divides, this has exponential distribution with mean 1/(*β_m_m*). The time *T_n_* until the next normal cell divides is similarly exponential with mean time 1/(*β_n_n*). Then, if we know we have a cell division at some point in time, we would like to know which type of cell will divide first. Specifically, we require
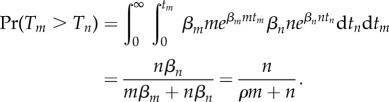

Thus, we just have to weight the mutant count by the relative increase in division rate. In particular, if we have *m* mutant cells and *n* − 1 non-mutant cells, then the probability that we have *m* mutants and *n* non-mutants after the next cell division requires a non-mutant to divide without a new mutation forming. This occurs with probability (*n* − 1)/(*n* − 1 + *ρm*)*μ*_0_. Similarly, if we have *m* − 1 mutant cells and *n* non-mutant cells, then the probability that we have *m* mutants and *n* non-mutants after the next cell division requires a mutant to divide, or a non-mutant to divide with a new mutation forming. This occurs with probability *ρ*(*m* − 1)/(*ρ*(*m* − 1) + *n*) +(*n*)/(*n* + *ρ*(*m* − 1))*μ*_1_. The recurrence is a statement of conditional probability connecting these two observations. ▪

The recurrence can be used to derive the probabilities 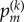 and the moments. However, an application of the generating function approach of theorem 3.2 to derive an analogous formula proved difficult. An example of the distribution from theorem 3.4 can be seen in figure 5*c*, where we have mutation rate *μ*_1_ = 0.05 and relative fitness *ρ* = 2. This gave a comparable distribution to figure 5*b*, where the mutation rate is *μ*_1_ = 0.20 with neutral relative fitness *ρ* = 1, although the variance is notably higher in figure 5*c*.

## 4. Distributions of subclones in mutated colonies

**Figure 6.**
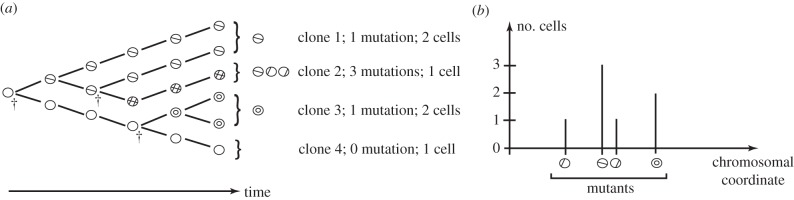
(*a*) A representation of clonal mutant growth; six cells result from five cell divisions, three of which produce four mutations (†), which cluster into four clones. (*b*) Representation of the cellular count for each mutation against illustrative chromosomal coordinates.

In the questions considered in §3, we just have the binary status of mutated or non-mutated. This is generally the status of a gene, or a portion of a chromosome that may be of interest, but could also be the status of a single nucleotide of DNA, which number in the billions. DNA sequencing techniques now mean that individual mutations can be distinguished by their position in the genome. For example, in figure 6*a*, we see that five of six cells are mutant, arising from four mutations produced during three cell divisions (†), that combine into four distinct clones. In figure 6*b*, we have the distribution of the number of cells for each mutation. This is a symbolic representation of the mutation and sequencing depth information obtained from modern experiments and points to other avenues of investigation. First, we would like to know the number of cells containing a randomly selected mutation. Second, we would like to know the number of clones. Third, we would like to know the number of cells in a randomly selected clone. Finally, we would like to know the number of distinct mutations in a randomly selected clone. We have the following results.

### 4.1. The number of cells containing a specific mutation

We have the following result for the first question.

**Theorem 4.1.**

*If* 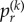 *is the probability that a randomly selected mutation exists in r cells in a colony of k cells, then we have*
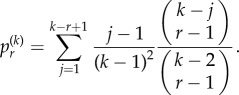

This differs slightly from the original problem considered by Luria and Delbrück in that instead of asking how many cells contain a mutation in a specific gene (or region), which may involve many different mutation events, we randomly sample a mutation from all mutations found in that region, and count the corresponding number of cells containing that mutation. We assume each mutation arises only once, which may not be true for large colonies or small genomes.

*Proof.* Now, there are *k* − 1 divisions that take place to give a sample size of *k*. Now, if we randomly select a mutation, it can arise during any of these divisions with equal probability. We let 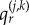 denote the probability that if a mutation forms when there are *j* cells, it is present in *r* cells when the cell population is *k* ≥ *j*. Then, if 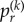 is the probability a randomly selected mutation is in *r* cells when the population is of size *k*, we have
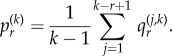

If the mutation arises when the population has size *j*, then this mutation may be present in any of 1 to *k* − *j* + 1 cells when the population size is *k*, depending on whether the cells containing the mutation divide. Thus, *j* ≤ *k* − *r* + 1. Furthermore, following a population size of *k* − 1, we either have *r* − 1 copies of the mutation and the next cell division duplicates a copy, or we have *r* mutant cells, and the dividing cell does not contain the mutation of interest. This gives us the recurrence
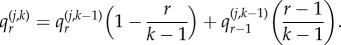

Now, if we start with the initial value 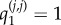, so that initially one of *j* cells carries the mutation, then we can show by substitution that this recurrence and initial condition is satisfied by the following expression:
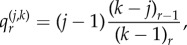
where (*a*)*_b_* = *a*(*a* − 1) … (*a* − (*b* − 1)) is the Pochhammer symbol. Substituting into the expression above then gives
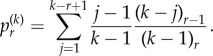

This is equivalent to the expression in the theorem. ▪

### 4.2. Distribution of the number of clones

The second problem requires the distribution of the number of clones. Every time a new mutation occurs, it will occur in a single cell that belongs to some clone already present. That cell will divide into two daughters, one of which will contain the new mutation. That cell will have a new combination of mutations and a new clone is born. We thus trivially observe that the number of clones is always one more than the number of cell divisions that produce new mutations. Now, mutations can arise during a cell division. For a colony of size *k*, we have *k* − 1 independent cell divisions in total, each of which may generate new mutations with probability *μ*_1_. We thus find the following.

**Theorem 4.2.**

*If C represents the number of clones, then we find that for a total population size k, C−*1* has binomial distribution Bin(k − *1*, *μ*_*1*_*).

### 4.3. Size distribution of mutant clones

The third question concerns the size of the clones. For example, in figure 6*a*, we note that clone 2 was formed in the third cell division, and contains a single cell. The associated distribution for the size of a random clone is described in the following result.

**Theorem 4.3.**

*Let* 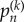 *represent the probability a randomly selected clone contains n cells, given a total population of k cells. Let* 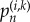 *be the corresponding probability for a clone formed in the ith cell division. Then,* 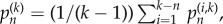 *where*
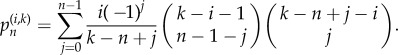

*Proof.* A new clone arises whenever a mutation occurs. For a population of size *k*, a randomly selected mutation arises with equal probability 1/(*k* − 1) at any of the *k* − 1 divisions that have taken place.

Let us suppose the clone appears at division *i*. We thus have 1 cell in the clone and *i* other cells. We let *r* = 0, 1, … *k* − 1 − *i* index the remaining divisions and 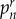 represent the probability of having *n* clonal cells after the cell division with index *r*. We thus have initial condition 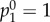. If we have *n* clonal cells after the cell division with index *r* (resulting in *r* + *i* + 1 cells in total), then the next division is a clonal cell with probability *n*/(*r* + *i* + 1). Conditioning over a single division then results in the recurrence
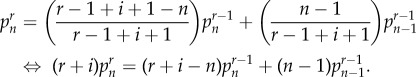

If we introduce the generating function 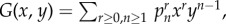 then substituting the recurrence results in the partial differential equation
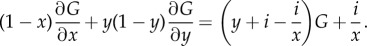
We then solve this using the method of characteristics with boundary condition *G*(0, *y*) = 1 to give
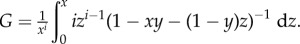

Three binomial expansions inside the integral then allow us to write *G* as a power series in *x*, *y* with coefficient
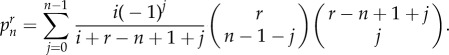
Substituting *r* = *k* − *i* − 1 then gives the desired form. ▪

### 4.4. Number of mutations in a random clone

Finally, we need the number of mutations in a randomly selected clone. For example, note that clone 2 from figure 6*a* is composed of three mutations, two of which formed during the third cell division. In general, we have the following result.

**Theorem 4.4.**

*Let X_i_ be the Bernoulli variable with success probability* 1/(*i* + 1) *for* *i* = 1,2,…, *k* − 1. *A clone arises at cell division i with probability* 1/(*k* − 1)*, where k is the total population size. The number of mutations accumulated by a clone formed in cell division i is* 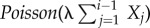, *where e^−*λ*^ = *μ*_0_*.

*Proof.* New mutations occur during any cell division with a probability *μ*_1_ = 1 − *μ*_0_. Now, if we assume that different mutations arise independently, then we can assume they are Poisson distributed per cell division with some parameter *λ* so that *μ*_0_ = *e^−*λ*^*. Now, if a clone occurs at division *i*, then any subsequent mutations form new clones and do not belong to this clone. However, any earlier mutations may have been incorporated into its lineage. If the first cell division has a mutation, then it occurs in this lineage with probability 1/2, the second division with probability 1/3, the *r*th with probability 1/(*r* + 1). The total number of mutations in the lineage is then a sum of identical Poisson variables over cell divisions in this lineage. ▪

## 5. Conclusion

We have shown that the number of mutated or proliferating cells in a clone has a natural dependency upon the total clone size, rather than time taken for a single cell to grow into the observed clone. This corresponds to the embedded Markov (or jump) chain of the continuous process, and combinatorial and generating function approaches can reveal their distributions.

The utility of these techniques has been demonstrated for epithelial tissue, where the relative likelihoods of different types of cell division were estimated. This is a model where different cell fates are the main difficulty. We also demonstrated for the pure birth process that different cell division rates can also be examined using these techniques. However, some situations may involve both complications, and derive from different models. Intestinal epithelium, for example, has different cell division rates, and the colonic crypts have a distinct model of tissue homeostasis. Each individual case will require its own separate analysis of the underlying jump process. Exploring these methods across the full range of tissue and/or mutation types is beyond the scope of this paper. However, we have provided sufficient examples to demonstrate that the general approach described is likely to be worth exploring in other scenarios.

The method described makes no assumption about dynamics, and can resolve population asymmetry (*a* = *c* in figure 1*b*), invariant asymmetry (*a* = *c* = 0) or imbalanced fate tilted towards proliferation (*a* > *c*) or differentiation (*c* < *a*). The clones can be set in a homeostatic or non-homeostatic tissue or indeed in a cell culture system. A requirement for the analysis shown in figure 1 is that terminally differentiated cells are not lost from the system by apoptosis or shedding. However, these processes can be accommodated if additional information such as the rate of cell loss is known. The scope of the method extends to all systems where cell fate is intrinsic rather than being regulated by spatial constraints such as in the intestinal crypt.

We have shown how this method can resolve the probabilities of each division outcome in small colony forming cells in primary human keratinocyte cultures [44]. This is likely to see applications to mutant keratinocytes such as resolving the imbalance in fate seen with keratinocytes harbouring p53 mutations under UV exposure [11].

This method can be applied to early time point data from *in vivo* lineage tracing experiments such as those also reported in [1,46] or analysing the dynamics of small p53 mutant clones. However, the time-independent approach relies on cells not being lost from the tissue. In the epidermis, once the surface is breached by differentiating cells, cell loss complicates the analysis. Intestinal epithelium is complex and highly dynamic. The location of stem cells within the crypt is key to the regulation of homeostasis, and live imaging has been required to resolve that not all Lgr5+ stem cells are functionally equivalent as was previously thought [47]. Our model does not address spatial aspects and is not suited to lineages such as enterocytes which are rapidly lost from the epithelium. Our method could be used to investigate Paneth cell precursors in a clonal frequency lineage tracing experiment *in vivo* or in organoid cultures, as differentiated Paneth cell turnover is slow [48]. However, to date, there are no published datasets suitable for such analysis.

In addition in the second part of the paper, we apply these insights to an emerging problem, the analysis of the frequency of mutations within a sample where the age of the constituent clones is not known. Such data are being generated by deep genomic sequencing studies of tumours, for example, and methods such as the time-independent analysis we present here are needed to help interpret the data.

The approaches discussed are exact but can be difficult to handle for large samples sizes and some asymptotics would be useful. Furthermore, the results all assume that the processes of cell division are Markovian, and so the cell cycle exponentially distributed. This is unlikely to be accurate, with cell cycle generally being better approximated by gamma distributions. This may have significant effects on some results and warrants further exploration.

## Funding statement

We acknowledge support from the Cambridge Cancer Centre Research Fellowship (A.R.), and the Medical Research Council (P.H.J.).

## Appendix A

Alternative proof of corollary 2.2 using stochastic processes methods.

**Proof.**

Consider a random walk that moves up or down by one unit at each step, starting from height 1. We are interested in the number of steps taken until we first reach height 0.

We let *u_n_* denote the probability of being at height 0 after *n* steps, where the walk is initially unrestricted and may move below or above height 0. This requires *x* up steps and *x* + 1 down steps for some *x* ≤ (*n* − 1)*/*2 and so we obtain the multinomial sum for *n* ≥ 1:
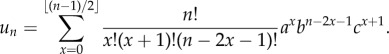

This can be used to construct an associated generating function:



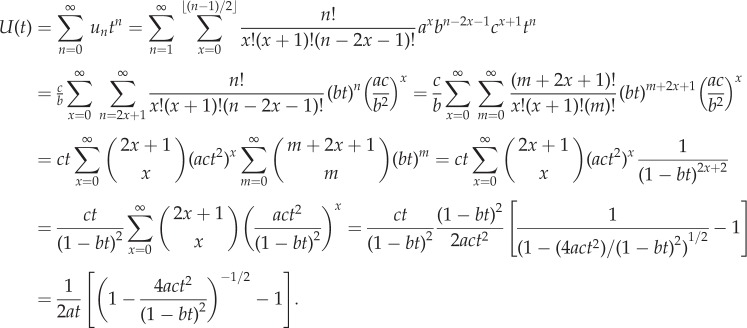


Here, we have used the identity 
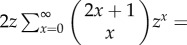
 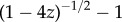 on the penultimate line.

Similarly, we let *v_n_* denote the probability of being at height 0 after *n* steps, this time starting from height 0. Again, we do not prohibit negative heights. This requires *x* up steps and *x* down steps for some *x* ≤ *n*/2, and so we obtain the multinomial sum for *n* ≥ 1:
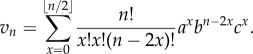


This also has an associated generating function:



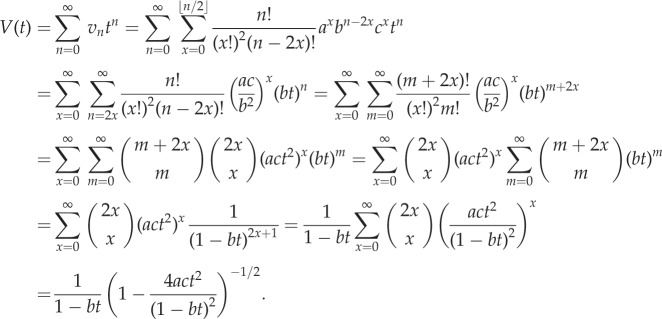


We are interested in the first visit to height 0 starting from height 1. Now, if we know we are at height 0 after *n* steps, then there must be a first visit to height zero after *r* steps for some *r* with 1 ≤ *r* ≤ *n*. If *f_r_* represents the probability of a first visit to 0 after *r* steps, then we then have the discrete convolution
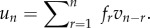


Multiplying by *t^n^* and summing then results in the following relation between generating functions:
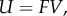
where 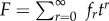 is the generating function for the probabilities *f_r_* we desire. Then, substituting the generating functions above yields the following:
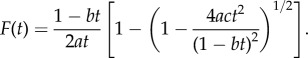


To obtain the required expression in corollary 2.2, we note that the generating function 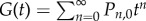 relates to the probability of ruin *P_n_*_,0_ when there are *n* cells present. We start from 1 cell, so this involves *n* − 1 steps and we find *f_n_* *_−_*_1_ = *P_n_*. In terms of generating functions, we find *G*(*t*) = *tF*(*t*), which gives the desired form for *G*(*t*). ▪
